# Infantile Scurvy, or Barlow's Disease

**Published:** 1910-05-21

**Authors:** 


### Diseases of Children.

### INFANTILE SCURVY, OR BARLOW'S DISEASE.

We recently published a letter from a correspondent concerning the relation, or rather the absence of relation, between rickets and infantile scurvy. It used to be stated that the bones of rickety children are so tender that the weight of the bedclothes upon the limbs becomes unbearable and the infant kicks off its coverings during sleep; it is now more generally contended that tenderness of the bones is not a sign of rickets at all, but that it is due to sub-periosteal hæmorrhages, which are produced by changes in the blood similar in nature to those of scurvy in adults. The following notes upon infantile scurvy, first clearly defined by Barlow, and therefore often spoken of as "Barlow's Disease," may be of interest.

The essential features of infantile scurvy would seem to be the presence of sub-periosteal blood extravasations; during the period preceding the eruption of the teeth these hæmorrhages may occur solely in the sub-periosteal region. Those who are impressed with the spongy voluminous bleeding gums of adult scurvy might be loth to diagnose infantile scurvy unless a similar condition of the gums were present here also, but this would seem to be by no means an essential part of the disease. As a matter of fact, the earlier authors describe the sub-periosteal hæmorrhages as occurring particularly in rickety children, but others do not admit any causal relation with rickets at all, though the two may occur at the same time. Both may be due to the same errors of diet. If, however, Barlow's disease consists simply in a hæmorrhagic form of rickets, it is difficult to explain why it is rare in certain countries where rickets is very common, and *vice versâ*. In America infantile scurvy is, or used to be, relatively frequent, to judge by the published statistics; rickets, on the contrary, is relatively rare. In Australia, whence numbers of papers upon rickets emanate, infantile scurvy is exceptional. Rickets, moreover, is particularly common amongst the poor, whereas infantile scurvy is most often seen amongst the middle classes and rich. In autopsies upon cases that have conformed with the clinical description of Barlow's disease with both sub-periosteal and subcutaneous hæmorrhages, the histological lesions of rickets have been found absent. In addition to all these points there can be little doubt that infantile scurvy often occurs in children who present no signs of rickets whatever.

Stress has been laid by some observers upon the rarity of hæmatomata in adult scurvy, although they are almost essential parts of the infantile type; as a matter of fact, however, hæmatomata, both sub-periosteal and otherwise, may occur in a marked degree of true scurvy. It is a remarkable feature of infantile scurvy that lesions of the gums may be entirely wanting, but it is equally noteworthy that gingivitis is absent just as much in toothless adults suffering from scurvy. The subcutaneous hæmorrhages are not essential parts either of infantile or adult scurvy; as evidence of the two being of the same kind may be mentioned the improvement which follows the administration of anti-scorbutic remedies, which is one of the most important facts in the whole range of medicine.

The reason why rickets and scurvy may coincide in some patients is because both are due, directly or indirectly, to errors of diet; the commonest such error is the employment of sterilised milk or prepared foods in place of cow's milk and fresh foods. Infantile scurvy, like the scurvy of adults, is a pathological state of the blood and of the tissues, whose cause is in the first place alimentary. It is characterised by a marked and progressive anæmia, a tendency to fainting attacks, an earthy complexion, marked muscular weakness, prostration and apathy, a fungous state of the gums in some of those who have teeth, and hæmorrhages in and on various organs, particularly beneath the skin, beneath the periosteum of bones, and in the muscles—more marked in the lower limbs than elsewhere. The malady is a consequence of the withholding of fresh foodstuffs; in adults it is due to privation and want of fresh meat and fresh vegetables; in the child that which is lacking is fresh milk or some other fresh food; in the child, as in the adult, scurvy is immediately benefited and soon cured by a return to those fresh foods which have been lacking. Rickets and infantile scurvy may coincide, but they are both due to a common cause rather than the one being the cause of the other. Tenderness of bones is a feature of infantile scurvy rather than of rickets, and this tenderness is due to sub-periosteal hæmorrhages, which may be the sole evidence of infantile scurvy in the milder cases, which are by no means uncommon.

